# Unpleasant Odors Affect Alerting Attention in Young Men: An Event-Related Potential Study Using the Attention Network Test

**DOI:** 10.3389/fnins.2021.781997

**Published:** 2021-12-07

**Authors:** Minggang Zhang, Xinyu Gong, Jiafeng Jia, Xiaochun Wang

**Affiliations:** School of Psychology, Shanghai University of Sport, Shanghai, China

**Keywords:** unpleasant odor, attentional cognition, attention network, ERP, ANT

## Abstract

Attention to unpleasant odors is crucial for human safety because they may signal danger; however, whether odor concentration also plays a role remains debated. Here, we explored the effects of two concentrations of pleasant and unpleasant odors on the attention network, comprising the alerting, orienting, and executive control networks. Behavioral responses were examined using the Attention Network Test, while electrophysiological responses were examined by assessing N1 and N2 amplitudes in 30 young men. We found that irrespective of odor concentration, an unpleasant odor induced larger cue-related N1 and N2 amplitudes in the alerting and executive control networks at occipital and frontal electrode sites and that was only paralleled by a reduced behavioral response time of cue-related trails in the alerting network. Thus, our results do not provide supporting evidence for a concentration-dependent effect, but they do suggest that more attentional resources are allocated to alerting-relevant stimuli to improve behavioral response times to a potential threat in young men.

## Introduction

Odor cues in the environment are often associated with potentially dangerous information, such as toxic gases or decayed food (Boesveldt et al., 2010; Ruser et al., 2021). Being aware of odor-related hazards timely aids in avoiding or escaping a source of danger, and paying attention to these odor cues is critical to human survival (Fanselow and Lester, 1988). Therefore, the present study aims at exploring whether the external odor stimuli will affect the attention function of human in an unknown and dangerous situation.

Attention is a basic cognitive ability in humans, and almost all aspects of human behavior are closely related to attentional processes (Ilmberger et al., 2001). In recent years, most studies exploring attention have been based on the attention network system theory put forward by Posner and Petersen (1990). Specifically, the attention network is divided into three separate but unified functional networks: the alerting network, the orienting network, and the execution control network (Posner and Petersen, 1990). The alerting network maintains a state that is sensitive to external stimuli and is ready to respond to these stimuli. This network is associated with the norepinephrine/locus coeruleus system and involves the main areas of the frontal cortex and the dorsal visual pathway leading to the parietal lobe. The orienting network selects attentional information from that obtained by the senses to direct attention to the location of a relevant cue. This network is associated with the superior parietal cortex, the temporal-parietal junction, and the frontal eye fields. The executive control network assesses conflict between target stimuli and distractors and involves high-level cognitive processes, such as conflict processing, inhibitory control, and decision-making. This network is mainly associated with the anterior cingulate cortex and the dorsal lateral prefrontal cortex (Posner and Rothbart, 2007; Petersen and Posner, 2012). And more recent studies suggest that the attention processing is related to central executive network (CEN), which includes the lateral prefrontal cortex, the parietal cortex, and the insula (Dixon et al., 2014; Fossati, 2019). Meanwhile, the neurocircuit involved in olfactory odor pleasantness processing are overlapped with the attention network in some extent. Ruser et al. (2021) summed up the central brain regions are more involved in odor pleasantness processing, and they show that the activation patterns of unpleasant odors (such as the BOLD signal) are stronger than those of pleasant odors. Specifically, the amygdala, the piriform cortex and other brain regions, and even a joint activation pattern network was created between the right piriform cortex, the left insular cortex, the orbitofrontal cortex and the anterior central gyrus. Sorokowska et al. (2016) also suggested a core network is comprised for odor pleasantness processing, which consists of the bilateral cingulate gyrus, the left middle frontal gyrus, the right middle frontal gyrus/lateral OFC, the bilateral parahippocampal gyrus, the right lentiform nucleus, the lateral globus pallidus, the right medial frontal gyrus/medial OFC, the left superior frontal gyrus and the right insula.

The pleasantness of odors plays important roles in human cognition, behavior, and emotion (Holland et al., 2005), and the pleasantness of odors has demonstrated effects on shifting visuospatial attention (Rinaldi et al., 2018), enhancing alertness (Warm and Dember, 1991; Shimizu et al., 2008), improving attention accuracy or task efficiency (Scholey et al., 2008; Liu et al., 2019), and attentional wayfinding (Hamburger and Knauff, 2019). However, the meta-analysis study demonstrated that the studies of olfactory odor processing leading a study bias which are more based on pleasant odor stimuli and few studies taking a contrast study between pleasant odors and unpleasant odors (Zou et al., 2016). Nonetheless, in previous related studies, unpleasant odors showed superior processing characteristics compared with pleasant odors. For example, the fMRI study demonstrated that the amygdala showed greater BOLD signals for unpleasant odors compared to pleasant odors, and the core structure of the primary olfactory cortex and the piriform cortex also showed greater activation patterns for unpleasant odors than pleasant odors (Jin et al., 2015; Sorokowska et al., 2016). Behavioral studies have shown that unpleasant and pleasant odors are responded to differently, with unpleasant odors typically detected faster and with greater accuracy (Bensafi et al., 2002a; Jacob and Wang, 2006; Boesveldt et al., 2010). Event-related potential (ERP) studies have shown that compared with the pleasant odor vanillin, the unpleasant odor hydrogen sulfide enhances the amplitude of the N2 component, and hydrogen sulfide odor is associated with shorter N1 and P2 latencies than those for a pleasant peach odor, together suggesting that unpleasant odors may attract more attentional resources (Kobal et al., 1992; Croy et al., 2013). On the other hand, besides the pleasantness of odors, the odor concentration is also an important aspect that should be considered in odor studies. However, the effect of odor concentration on behavior and neurophysiology is still under debate. Although several ERP studies have shown that the amplitudes of the early and late components of cortical somatosensory evoked potentials increase with increasing odor concentration (Pause et al., 1997; Tateyama et al., 1998), other studies have reported that the behavioral responses to strong and weak concentrations of unpleasant and pleasant odors do not show a difference in response time (Bensafi et al., 2002a; Croy et al., 2013).

Above these, previous studies have shed light on the effects of odor pleasantness and odor concentration on attentional behavior and brain processing partly. However, we believe that there are two issues that need to be re-emphasized. First, considering that both odor pleasantness and odor concentration can affect odor processing efficiency, conclusions drawn from a single investigation into the effects of odor pleasantness or odor concentration are limited. However, we found that most of the previous studies only investigated one aspect of odor pleasantness or odor concentration, while few studies combined the two factors to investigate simultaneously. Therefore, it is necessary to investigate the effects of odor pleasantness and odor concentration simultaneously. Second, paying attention to odor cues in the environment is important for human safety, but the effects of odor pleasantness and concentration on attention have been investigated only partly or indirectly. In other words, there has been no systematic study of the relationship between odor stimuli, including pleasantness and concentration, and the attention network using behavioral and ERP indicators, more specifically, by measuring the efficiency of the three attention networks using the Attention Network Test (ANT, see Methods section “Attention Network Test” for a detailed description of ANT). Therefore, we need to further understand the relationship between attention function and odor processing.

The ERP reflects synchronous neural activity associated with specific cognitive events, providing a record of voltage fluctuations in the scalp. ERPs offer time resolution in the millisecond range and allow the description of continuous internal processes that behavioral measurements cannot provide (Yang and Xiang, 2019). ERPs describe time-dynamic information of the neural network of attention, while behavioral measurements obtained using ANT assess the efficiency of the attention network. Previous studies using ANT and ERPs have shown that the performance of the alerting network and the orienting network are closely related to the cue-locked N1 potentials, whereas the performance of the executive control network is related to the target-locked N2 component (Neuhaus et al., 2010; Kratz et al., 2011; Donohue et al., 2016; Williams et al., 2016; Yang and Xiang, 2019).

The N1 component appears approximately 150–250 ms after a stimulation is presented, reflecting early visuospatial processing of the visual cortex (Wascher et al., 2009; Neuhaus et al., 2010). An increase in the amplitude of N1 is closely related to effective cue stimulation (Nobre et al., 2000). For example, an ERP study combined with ANT has shown that the amplitude of N1 changes significantly in the alerting and orienting networks: when no cue is provided in ANT, the N1 amplitude is significantly lower than when a double cue is given; and when a spatial cue is given in ANT, the N1 amplitude is lower than when a center cue is provided (Neuhaus et al., 2010).

The N2 component is distributed primarily in the frontocentral electrode sites. Its amplitude peaks between approximately 250 and 350 ms after the target stimulus is presented and is thus considered to be associated with monitoring processes or with conflict resolution in trials with correct responses (Kopp et al., 1996; Ridderinkhof et al., 2004; Bartholow et al., 2005; Larson et al., 2014). In ERP studies that used ANT, the N2 amplitude in the incongruent target condition is more negative than that in the congruent target condition (Larson et al., 2014; Yang and Xiang, 2019). The theory of conflict monitoring holds that anterior cingulate cortex (ACC) is the basic mechanism underlying the N2 component (Veen and Carter, 2002). Studies of source location also support that the fronto-central N2 potential is able to generate from ACC (Ladouceur et al., 2007). When a conflict occurs, more endogenous attention resources are recruited to improve resolving the conflicting stimuli (Larson et al., 2014; Groom and Cragg, 2015).

N1 and N2 components, as classical components in visual or auditory attention studies, also play a role in olfactory odor processing studies (Tang et al., 2019; Fallon et al., 2020). In the study of healthy people, the amplitude of N1 enhanced with the increase of odor concentration (Turetsky et al., 2003), and in the clinical study of schizophrenia, the amplitude of N1 is more significant in the presence of negative odor in people with schizophrenia compared to in healthy people (Pause et al., 2008; Kayser et al., 2010). The N2 component shows larger amplitude in the mid-frontal and the left frontal-temporal areas under longer odor stimuli (500 ms) compared to shorter odor stimuli (300 ms) (Tang et al., 2019), and a significant olfactory-visual interaction is presented in the N2 component in the central-frontal electrodes of odor-face ERPs (Cook et al., 2017).

The present study takes advantage of the time resolution provided by assessing electrophysiological responses through ERPs and investigates behavioral responses using ANT to determine the effects of the pleasantness of odors at different concentrations on the attention network comprising alerting, orienting, and executive control.

Guided by previous studies on odor pleasantness and odor concentration, since the most unpleasant odors are more likely to signal potential hazards to the persons, in present study, We hypothesized that unpleasant odor would show greater behavioral performance and stronger activation patterns in the alerting, orienting, and executive control networks compared to pleasant odor, especially in the alerting network. Furthermore, according to the debated conclusions of odor concentrations, we hypothesized that the neurophysiological effects would be greater as odor concentration increased but that would not be accompanied by behavioral response differences among the alerting, orienting and executive control networks.

## Materials and Methods

### Participants

In total, 30 male undergraduate students between 18 and 30 years of age (mean, 23.12 years; standard deviation, 2.3 years; self-reported Chinese race/ethnicity) from Shanghai University of Sport were recruited for this study through campus posters and were randomly assigned to one of two groups (weak or strong odor concentration). Eligible participants were right-handed, had no history of neurological or mental illness that could potentially affect the results of the experiment, and were in good physiological condition, especially having good function of the olfactory system. The “sniffin sticks” method is used to test the olfactory function. This method consists of three sub-tests, which including olfactory threshold, olfactory discrimination and olfactory identification (TDI), and adding up the scores on the three sub-tests to obtain a total TDI score, a total score of more than 30 indicates good olfactory function. All participants had TDI scores above 30 (mean, 34.52; standard deviation, 3.7), indicating that their olfactory function was normal (Hummel et al., 2007). This study was reviewed and approved by the ethics review committee of Shanghai University of Sport. Participants were informed of their experimental rights and obligations, and they provided informed written consent obtained in a manner consistent with the Declaration of Helsinki to participate in this study. Participants were compensated 100 RMB (approximately $15) after completing the experiment.

### Attention Network Test

The Attention Network Test (ANT) was developed by Fan et al. (2002). ANT combines cued target detection (Posner, 1980) with distracting flanker displays (Eriksen and Eriksen, 1974) to evaluate the efficiency of the attention network. The behavioral efficiency of all three attention networks can be demonstrated via the response time (RT) of participants in this task. Specifically, Alerting = RTno cue-RTdouble cue;Orienting = RTcenter cue-RTspatial cue;Executive control = RTincongruent-RTcongruent (Fan and Posner, 2004; Neuhaus et al., 2010; Yang and Xiang, 2019).

**FIGURE 1:**
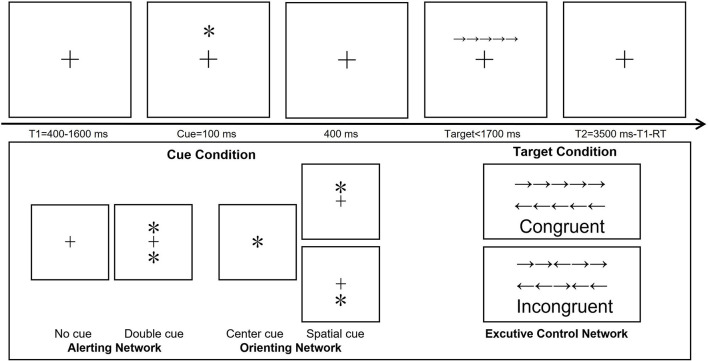
Schematic diagram depicting the Attention Network Test paradigm. T1, represents the fixation time; T2, the interval between two trials; RT, response time. ^∗^Means the cue types in the altering and orienting networks of ANT.

ANT (Fan et al., 2002) was conducted via the E-Prime 2.0 software package (Psychology Software Tools Inc.) and presented on a 17-inch Lenovo personal computer monitor with a white background that was located approximately 80 cm in front of the participants. The ANT paradigm used in this study is shown in Figure 1. In a trial of ANT. First, a fixation with a random variable time of 400–1,600 ms is presented, and the variable period is used to generate attention uncertainty regarding the appearance of the cues. Then, a warning cue was presented for 100 ms. After the warning cue was presented, there was a short fixation interval of 400 ms. Then, participants responded to the presented target Flanker task with a maximum reaction time (RT) of 1,700 ms. After the participants responded, the target Flanker task disappeared immediately and a post-target fixation (3,500-duration of first fixation-RT) was presented. Thus, the total duration of each trail is 4,000 ms. Specifically, in alerting and orienting measurement, there are four kinds of cue conditions: no cue, double cue, center cue, and spatial cue. (1) no cue (fixation cross only); (2) double cue (one asterisk above and one asterisk below the fixation cross); (3) center cue (one asterisk in the center of the screen); and (4) spatial cue (one asterisk either above or below the fixation cross). The target stimulus consisted of a single row of five horizontal arrows. The participants were instructed to react quickly and accurately to indicate the direction of the arrow located in the center of the five arrows (the two arrows on either side of the center arrow were considered flankers). The target stimulus consisted of two conditions: the congruent condition (all five arrows faced the same direction) and the incongruent condition (the center arrow faced a direction opposite of the other four arrows, which all faced the same direction as one another). When the center arrow faced left, the participant used the left index finger to press the letter A on the keyboard; when the arrow faced right, the participant used the right index finger to press the letter L on the keyboard. The four cues were presented randomly and shown the same number of times. The congruent and incongruent target stimulus conditions were also shown the same number of times.

The experiment comprised two parts, a practice phase and the formal experiment. The practice phase consisted of 24 trials in which feedback was given on whether the participant’s response was correct or not after each trial. The formal experiment was conducted after the participant scored 95% correct in the practice phase. The formal experiment comprised four blocks, each of which consisted of 80 trials, for a total of 320 trials.

### Odor

The pleasant odor was rosemary essential oil (Sigma; Germany), and the unpleasant odor was ammonia (Chinese Medicine

Reagent; Shanghai, China). Each odor had two concentration levels. The strong concentration of rosemary essential oil had a purity > 99%, and the weak concentration was diluted 1:1 with 1,2-propanediol. Ammonia was diluted with plasma-treated water to 1.0% for the strong concentration and to 0.05% for the weak concentration (Covington et al., 1999; Bensafi et al., 2002a). The dilutions were carried out in a laboratory under the guidance of a professional chemist to ensure the precision of the odor concentrations. The diluted liquid (10 mL) was placed in four brown flasks (15 mL, 1.7 cm diameter at the opening, 5.8 cm high) and stored in a cool place to minimize volatility (Bensafi et al., 2002a). The fluids were replenished regularly to ensure consistency of the odor stimulation received by each participant. We recruited 12 male volunteers (right-hand dominant; mean age, 21.36 years; standard deviation, 1.9 years) who did not take part in the formal experiment to perform a preliminary examination of the concentration and pleasantness of each odor. They were asked to rate the odor concentrations on a 7-point Likert scale (1 being extremely weak, and 7 being extremely strong), and to rate odor pleasantness on a scale of −50 (extremely unpleasant) to 50 (extremely pleasant) (Croy et al., 2013). The flasks containing the liquids were placed 1 cm below both nostrils. Participants inhaled each scent for 1 s, with 30 s between odors (Bensafi et al., 2002a). The sequence of the odor presentation was counterbalanced using a Latin square design. This preliminary experiment showed that there was a significant difference in odor concentration scores for the same odor between the strong and weak concentrations, and a significant difference in odor pleasantness scores at both concentrations between the two odors, but there was no difference in concentration scores between the rosemary essential oil and ammonia odors at the same concentration.

### Event-Related Potential Acquisition and Computation

Electroencephalography (EEG) activity was recorded using Brain Amp equipment (Brain Products GmbH; Germany) with 64 channels. Electrodes are distributed according to the extended International 10/20 system. One electrode was placed above and one placed below the right eye, and two electrodes were placed lateral to the left eye to monitor the eye movements (vertical and horizontal electroencephalograms, respectively). The impedance of all electrodes was held below 10 KΩ. The EEG signal sampling rate was 1,000 Hz, with a bioamplifier filter using a band-pass of 0.1–250 Hz. Brain Vision Analyzer 2.1 (Brain Products GmbH) was used for EEG signal analysis. EEG signals were converted to average reference and off-line filtered with a 0.01–30 Hz (24 dB/octave) pass-band. EEG segments with artifacts exceeding ± 100 μV were removed. Data were then segmented relative to stimulus onset (200 ms pre-cue to 800 ms post-target, including a 500-ms pre-target interval). 200–100 ms pre-cue baseline correction was applied. Average EEG signals were calculated for each experimental condition and group to obtain the respective ERPs. A minimum of 30 artifact-free sweeps was averaged for each analyzed experimental condition, and only correctly responded trials were analyzed.

Average referenced ERP components N1 and N2 were determined semi-automatically with a visual control. Cue-locked N1 was examined to allow for exploratory analysis of N1 modulation by attentional top–down processing. The cue N1 (150–250 ms) and target N1 (650–750 ms) potentials both had waveforms involving the parietal (averaged P3 and P4 electrodes) and occipital (averaged PO3 and PO4 electrodes) sites (Yang and Xiang, 2019). N2 was observed as a prominent pleasant odor deflection between 250 and 350 ms post-target stimulus and was assessed at two midline electrodes, Fz and FCz (which are associated with the frontal-central ERPs), to allow for analysis of executive control (Williams et al., 2016). In the amplitude measurement of N1, which cue–locked ERP component were calculated by visual inspection of the grand -average waveforms Cue-N1 (150–250 ms) and target N1 (650–750 ms) averaged the amplitudes of the electrodes P3 and P4 in the parietal and the electrodes PO3 and PO4 in the occipital, respectively. And the amplitude measurements was considered frontal-centrally (FZ, FCZ) by averaging the values for the electrodes for N2 component (250–350 ms). The grand average ERP waveforms from all recorded electrodes were examined to create a topographic map.

### Experimental Procedure

Participants sat in a comfortable chair in a room that measured 5 m × 3 m × 3 m and was sufficiently ventilated to avoid the accumulation of odors. For stability and consistency with odor presentations, participants placed their head in a bracket located in front of them, with the height adjusted for each participant. The entire task consisted of three parts, that is, under three conditions, with ANT conducted the same for each: odorless (no odor presented), unpleasant odor presented, and pleasant odor presented. For odor presentations, the odor-filled flasks were placed by an investigator approximately 1 cm below the participant’s nostrils every 1 min for a 1-s inhalation, with timing controlled by the investigator. The participants were asked not to interact with the experimenter during the task (Diego et al., 1998; Bensafi et al., 2002a). The order in which the three conditions were presented was balanced using a Latin square design between participants to control for between-participant effects. The three experimental parts of ANT consisted of 12 blocks (960 trials in total) and lasted approximately an hour in total. Participants were given a 5-min break to counteract the effect of fatigue at the end of each odor condition and a 1-min break between blocks. At the end of the entire ANT task, each participant was asked to rate the intensity and pleasantness of the odors they received. The scoring criteria were consistent with those described above for the preliminary experiment.

### Statistical Analysis

#### Behavioral Analysis

To assess whether there were significant differences in odor concentration intensity and pleasantness scores, a 2 (odor concentration: strong vs. weak) × 2 (odor pleasantness: pleasant vs. unpleasant) mixed analysis of variance (ANOVA) was conducted. RT and accuracy of all cue and target conditions in ANT were analyzed with SPSS 22.0 for windows (Chicago, IL, United States). Specifically, only trials with correct responses with RTs between 200 and 1,500 ms were included in the further statistical analysis (Kratz et al., 2011). For assessing the alerting network, a 2 (odor concentration: strong vs. weak) × 3 (odor: odorless, unpleasant, pleasant) × 2 (cue: no vs. double) mixed ANOVA was conducted. For assessing the orienting network, a 2 (odor concentration: strong vs. weak) × 3 (odor: odorless, unpleasant, pleasant) × 2 (cue: center vs. spatial) mixed ANOVA was conducted. For executive control network, a 2 (odor concentration: strong vs. weak) × 3 (odor: odorless, unpleasant, pleasant) × 2 (target: incongruent vs. congruent) mixed ANOVA was conducted. For all those analyses, the odor concentration was treated as a between-group factor, whereas the cues, targets, and odor types were the within-group factors.

#### Event-Related Potential Analysis

The alerting network effect was calculated using a 2 (odor concentration: strong vs. weak) × 3 (odor: odorless, unpleasant pleasant) × 2 (cue: no vs. double) × 2 (site: parietal vs. occipital) mixed ANOVA for cue N1 and target N1 potentials. To assess the orienting network effect, a 2 (concentration: strong vs. weak) × 3 (odor: odorless, unpleasant, pleasant) × 2 (cue: center vs. spatial) × 2 (site: parietal vs. occipital) mixed ANOVA for cue N1 and target N1 potentials was conducted. To assesses attention processing of executive control, a 2 (odor concentration: strong vs. weak) × 3 (odor: odorless, unpleasant, pleasant) × 2 (target: incongruent vs. congruent) × 2 (electrode: Fz vs. FCz) mixed ANOVA for the N2 component was conducted. For those analyses, the concentration was treated as the between-group factor, whereas the cues, targets, electrode sites, and odor types were the within-group factors.

Both for the behavioral and ERP data analyses, the calculation of the mean numeric values including RT, ACC, and the ERP amplitudes were under cues and targets condition in the ANT, and the interaction effect between odor concentration and odor types was further analyzed in the three attention networks respectively. The significance level was set at 0.05. Gaussian distribution was assessed with the Kolmogorov-Smirnov test. Partial eta squared was computed to estimate the effect sizes of the main and interaction effects, and when the main effect of the factor was significant, *post hoc* analyses were computed using contrast analysis.

## Results

### Behavioral Response

**FIGURE 2:**
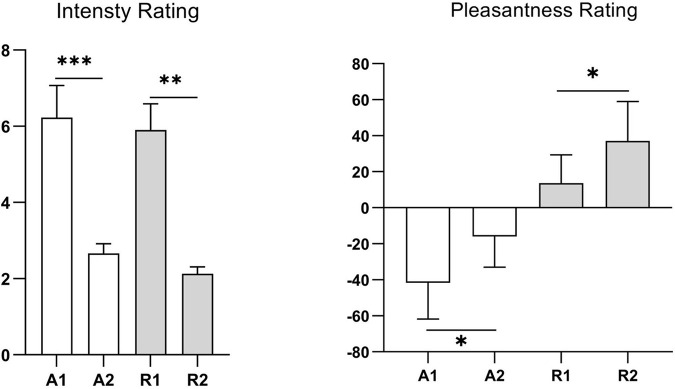
Odor concentration intensity and pleasantness ratings of participants in the formal study. A1, represents strong ammonia concentration; A2, weak ammonia concentration; R1, strong rosemary essential oil concentration; R2, weak rosemary essential oil concentration. Values represent the mean, and error bars indicate the standard error (SE). ^∗^*p* < 0.05; ^∗∗^*p* < 0.01; ^∗∗∗^*p* < 0.001.

The mean odor concentration intensity scores of the two concentrations (weak vs. strong) for each odor and the mean pleasantness rating scores of the two odors (rosemary essential oil vs. ammonia) were consistent with those in the preliminary test, indicating that the odor concentration and pleasantness levels were consistent among participants (Figure 2). The main effects of odor concentration and odor pleasantness were significant (*P* < 0.05), indicating that the strong and weak concentrations of each odor were well distinguished and that there was a significant difference in pleasantness between the two odors.

**FIGURE 3:**
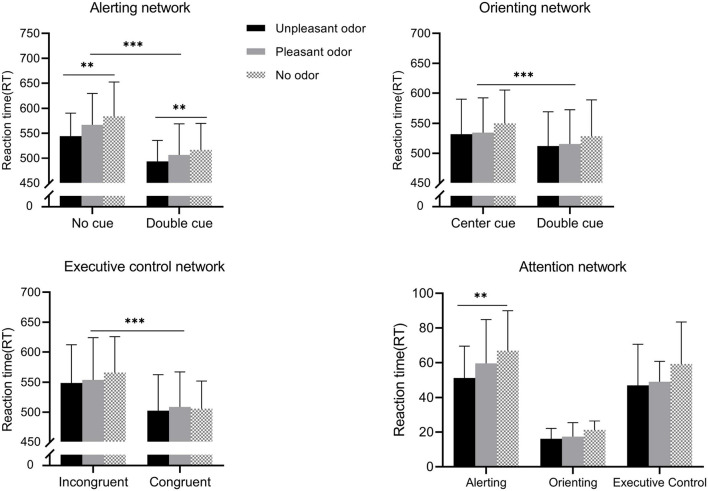
Reaction time (RT) for the different odor conditions for each attention networks. Values represent the mean, and error bars indicate the standard error (SE). ^∗∗^*p* < 0.01; ^∗∗∗^*p* < 0.001.

For participant RTs (Figure 3), the mixed ANOVA to assess the alerting network revealed significant main effects of odor and of cue conditions [odor: *F*_(__2_, _28__)_ = 7.09, *P* = 0.002, η*_*p*_*^2^ = 0.21; cue: *F*_(__1_, _28__)_ = 659.63, *P* < 0.001, η*_*p*_*^2^ = 0.93], and the interaction between odor and cue was also significant [*F*_(__2_, _28__)_ = 5.73, *P* = 0.005, η*_*p*_*^2^ = 0.17]. No other main effect or interaction of effects was significant (*P*-values > 0.05). The contrast test showed that the RT for the unpleasant odor response was the shortest (519.3 ± 10.9 ms), followed by the pleasant odor (537.1 ± 11.5 ms), and lastly no odor (550.5 ± 11.3 ms). The RT for the no-cue condition (565.3 ± 11.6 ms) was longer than that for the double-cue condition (506.2 ± 10.9 ms). Further analysis of the significant interaction revealed that the RT following the presentation of the unpleasant odor was faster than for the no odor or pleasant odor presentation in the double-cue condition but not in the no-cue condition.

The mixed ANOVA assessing the orienting network revealed a significant main effect of cue [*F*_(__1_, _28__)_ = 725.88, *P* < 0.001, η*_*p*_*^2^ = 0.96]. No other main effect or interaction of effects was significant (*P*-values > 0.05). The center cue (536.9 ± 11.2 ms) had a longer RT than the spatial cue (518.9 ± 11.1 ms).

The mixed ANOVA assessing the execution control network showed that the main effect of target was significant [target: *F*_(__1_, _28__)_ = 349.68, *P* < 0.001, η*_*p*_*^2^ = 0. 92]. No other main effect or interaction of effects was significant (*P*-values > 0.05). Incongruent target conditions (555.8 ± 12.3 ms) required longer RTs compared with congruent target conditions (504.1 ± 11.2 ms).

**FIGURE 4:**
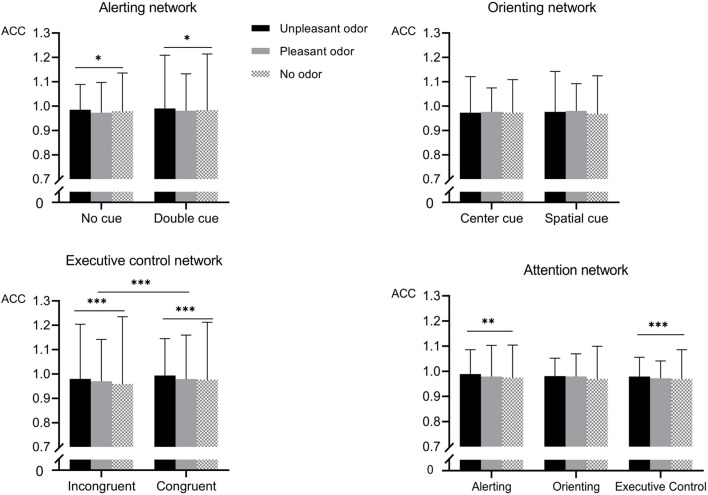
Accuracy (ACC) for the different odor conditions for each attention networks. Values represent the mean, and error bars indicate the standard error (SE). ^∗^*p* < 0.05; ^∗∗^*p* < 0.01; ^∗∗∗^*p* < 0.001.

For accuracy (Figure 4), the mixed ANOVA assessing the alerting network revealed a main effect of odor as well as a significant interaction between odor and cue [odor: *F*_(__2_, _28__)_ = 3.89, *P* = 0.02, η*_*p*_*^2^ = 0.13; odor × cue: *F*_(__2_, _28__)_ = 9.2, *P* < 0.001, η*_*p*_*^2^ = 0.25]. No other main effect or interaction of effects was significant (*P-*values > 0.05). The highest accuracy was found for the unpleasant odor (98.8% ± 0.8), followed by the pleasant odor (97.7% ± 0.9) and the odorless (97.4% ± 1.1) conditions. Follow-up analysis of the significant interaction revealed that accuracy following the presentation of the unpleasant odor was higher than for the no odor or pleasant odor presentation in the no-cue condition but not in the double-cue condition.

The mixed ANOVA assessing the orienting network detected no significant effects for accuracy (*P*-values > 0.05).

The mixed ANOVA assessing the executive control network revealed significant main effects for odor and for target conditions [odor: *F*_(__2_, _28__)_ = 35.54, *P* < 0.001, η*_*p*_*^2^ = 0.56; target: *F*_(__1_, _28__)_ = 286.15, *P* < 0.001, η*_*p*_*^2^ = 0.9] and a significant interaction between odor and target [*F*_(__2_, _28__)_ = 10.21, *P* < 0.001, η*_*p*_*^2^ = 0.28]. No other main effect or interaction was significant (*P*-values > 0.05). The accuracy in the congruent target conditions (98.2% ± 0.8) was higher than that in the incongruent target conditions (96.9% ± 1.3). The unpleasant odor conditions had the highest accuracy (98.7% ± 0.9), followed by the pleasant odor (97.4% ± 1.1) and the odorless (96.8% ± 1.4) conditions. Further analysis of the significant interaction revealed that the accuracy following the presentation of the unpleasant odor was higher than for the no odor or pleasant odor presentation in the incongruent targets but no in the congruent targets.

### Event-Related Potential Results

#### Alerting Network Effect (No Cue vs. Double Cue)

##### Posterior Cue N1 Amplitude

The mixed ANOVA results assessing the cue N1 amplitude indicated that the main effects of odor, cue, and site were significant [odor: *F*_(__2,__28)_ = 2.48, *P* = 0.04, η*_*p*_*^2^ = 0.12; cue: *F*_(__1,__28)_ = 4.93, *P* = 0.03, η*_*p*_*^2^ = 0.16; site: *F*_(__1,__28)_ = 14.36, *P* = 0.001, η*_*p*_*^2^ = 0.36]. The interactions of odor × site and of cue × site were significant [odor × site: *F*_(__2,__28)_ = 3.96, *P* = 0.02, η*_*p*_*^2^ = 0.13; cue × site: *F*_(__1,__28)_ = 6.15, *P* = 0.02, η*_*p*_*^2^ = 0.19]. No other main effect or interaction of effects was significant (*P*-values > 0.05). The cue N1 amplitude was larger for unpleasant odors (−2.4 ± 0.4μV) than for the odorless condition (−1.79 ± 0.6μV) or for pleasant odors (−1.68 ± 0.5 μV); the double-cue condition (−1.8 ± 0.3μV) also showed a more negative amplitude than the no-cue condition (−0.7 ± 1.2μV). The cue N1 amplitude at the occipital site (−2.65 ± 0.4 μV) showed a greater negative amplitude than at the parietal site (−1.89 ± 0.7μV). The follow-up analysis of the significant interaction between odor and site revealed that the cue N1 amplitude was greater for the unpleasant odor than for the odorless condition or the pleasant odor at the occipital site but not at the parietal site. In the double-cue condition, the cue N1 amplitude was also larger than that for the no-cue condition at the occipital site but not at the parietal site. These results suggested that participants showed higher alertness to upcoming stimuli under unpleasant rather than pleasant odor conditions and that the alerting state differed in different brain regions.

##### Target N1 Amplitude

**FIGURE 5:**
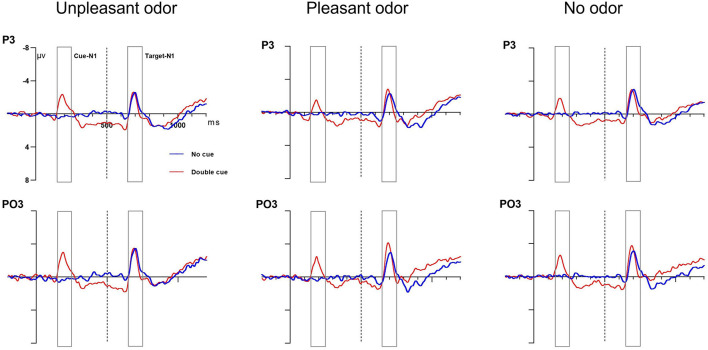
Grand average ERP cue amplitude data for the alerting network. No cue (blue lines) and double cue (red lines) conditions at electrodes P3 and PO3. Cue onset at 0 ms (solid line) and target onset at 500 ms (dashed line).

The mixed ANOVA results indicated that the target N1 amplitude for the cue-locked condition showed significant main effects for

the cue and for the site conditions[cue: *F*_(__1,__28)_ = 5.6, *P* = 0.02, η*_*p*_*^2^ = 0.18; site: *F*_(__1,__28)_ = 15.3, *P* = 0.001, η*_*p*_*^2^ = 0.37]. The interaction between cue and site was also significantly different [*F*_(__1_, _28__)_ = 4.46, *P* = 0.04, η*_*p*_*^2^ = 0.15]. No other main effect or interaction of effects was significant (*P*-values > 0.05). The occipital site (−3.81 ± 0.8μV) showed target N1 amplitudes significantly larger than the parietal site (−2.38 ± 1.4μV). The double-cue condition (−3.56 ± 2.1 μV) also had a significantly larger negative amplitude than the no-cue condition (−2.25 ± 1.7 μV). The follow-up analysis of the interaction indicated that for the occipital site, the target N1 amplitude was larger than that at the parietal site for the no-cue condition but not for the double-cue condition. Grand average ERP waveforms at electrodes P3 and PO3 in response to the alerting-related cue condition are presented in Figure 5.

#### Orienting Network Effect (Center vs. Spatial Cue)

##### Cue N1 Amplitude

The mixed ANOVA results for the cue N1 amplitude showed that the main effect of the cue condition was statistically significant and that the interaction between cue and site was also statistically significant [cue: *F*_(__1,__28)_ = 10.81, *P* = 0.003, η*_*p*_*^2^ = 0.3; cue × site: *F*_(__1,__28)_ = 7.07, *P* = 0.01, η*_*p*_*^2^ = 0.22]. No other main effect or interaction of effects was significant (*P-*values > 0.05). The spatial cue (−1.14 ± 0.2 μV) was associated with a larger negative amplitude than the center cue (−0.59 ± 0.3μV). The follow-up analysis of the significant interaction indicated that the cue N1 amplitude for the spatial cue was more negative than that for the center cue at the occipital site but not at parietal site.

##### Target N1 Amplitude

**FIGURE 6:**
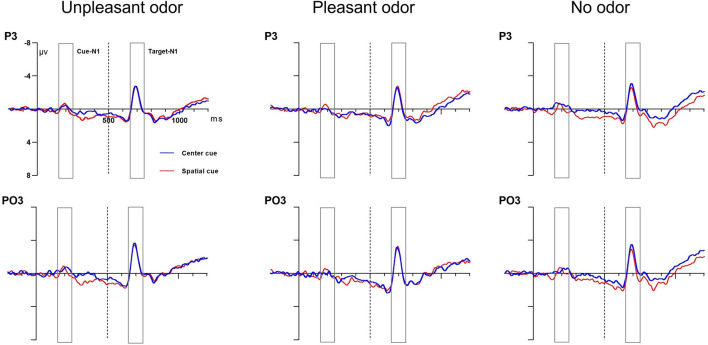
Grand average ERP cue amplitude data for the orienting network. Center cue (blue lines) and spatial cue (red lines) conditions at electrodes P3 and PO3. Cue onset at 0 ms (solid line) and target onset at 500 ms (dashed line).

The results of mixed ANOVA assessing the target N1 amplitude for the cue-locked condition found no main effect of cue, odor, concentration, or site or any significant interaction between these factors (*P*-values > 0.05). Grand average ERP waveforms at electrodes P3 and PO3 in response to the orienting-related cue condition are presented in Figure 6.

#### Executive Control Effect (Incongruent vs. Congruent Target)

##### N2 Component

**FIGURE 7:**
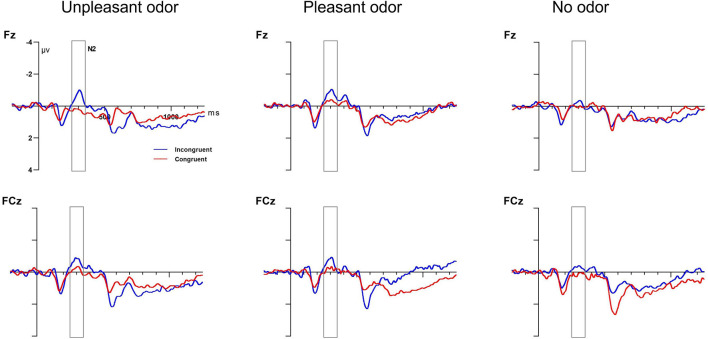
Grand average ERP target amplitude data for the executive control network. Incongruent target (blue lines) and congruent target (red lines) conditions at electrodes Fz and FCz. Target onset at 0 ms (solid line).

The mixed ANOVA results for the N2 amplitude indicated significant main effects for odor and target and a significant interaction between odor and electrode [odor: *F*_(__2,__28)_ = 0.63, *P* = 0.04, η*_*p*_*^2^ = 0.14; target: *F*_(__1,__28)_ = 1.51, *P* = 0.03, η*_*p*_*^2^ = 0.19; odor × electrode: *F*_(__1,__28)_ = 5.89, *P* = 0.005, η*_*p*_*^2^ = 0.19]. No other main effect or interaction of effects was significant (*P*-values > 0.05). The unpleasant odor was associated with the greatest N2 amplitude (−1.55 ± 0.7 μV), followed by the odorless condition (−1.27 ± 0.9 μV) and then the pleasant odor (−1.12 ± 0.6 μV). The incongruent target condition (−1.37 ± 0.2 μV) showed a larger negative N2 amplitude than the congruent target condition (−0.89 ± 0.4 μV). Further analysis of the significant interaction indicated that the N2 component showed a greater amplitude for the pleasant odor and for the odorless condition than for the unpleasant odor at the Fz electrode but not at the FCz electrode. Grand average ERP waveforms at electrodes Fz and FCz in response to target condition are presented in Figure 7.

**FIGURE 8:**
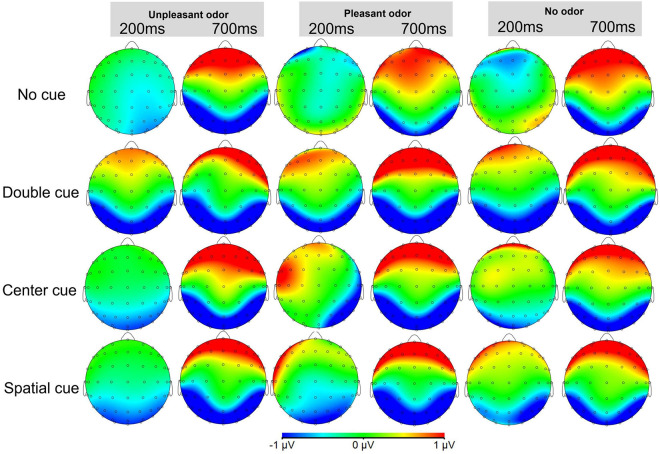
Topographic maps of the voltage differences in the grand-average ERP cue-N1 component (150–250 ms) and target-N1 component (650–750 ms) among the odor conditions.

**FIGURE 9:**
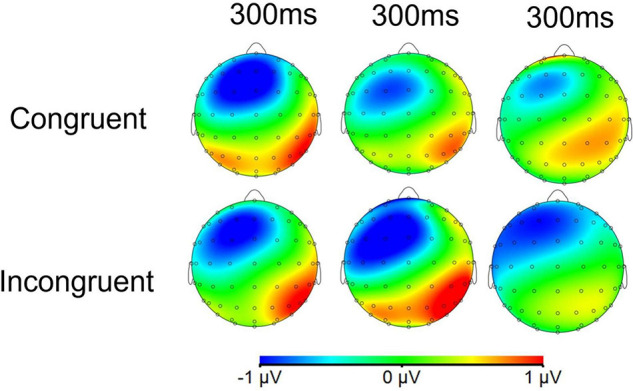
Topographic maps of the voltage differences in the grand-average ERP N2 component (250–350 ms) among the odor conditions. The three columns from left to right are unpleasant odor, pleasant odor and no odor conditions, respectively.

Topographic maps of the voltage differences in the N1 and N2 component amplitudes are presented for each condition in Figures 8, 9, respectively.

## Discussion

In this study, we assessed behavioral and electrophysiological responses to explore the effects of pleasant vs. unpleasant odors at two different concentrations on alerting, orienting, and executive control functions of the attention network. For this purpose, participants were randomly assigned to strong or weak odor concentrations group and perform the ANT under pleasant, unpleasant and odorless condition, respectively, while ERP data were acquired. Thus, the behavioral and ERP data were considered together. The present study suggests that, the unpleasant odor attracted more attention resources for assessing upcoming stimuli. Specifically, the combination of ANT behavioral response and ERP data showed that the alerting network enhanced more and attracted more attention resources compared with the orienting and the executive control network after the unpleasant odors was presented. By contrast, no difference was detected in behavioral or ERP responses associated with the concentration of the odor regardless of the pleasantness of the odor.

### Alerting Network and Odor Pleasantness

After the unpleasant odor stimulus, the N1 amplitude associated with the alerting-related cue presentation was significantly larger than after the pleasant odor or in the odorless condition, indicated that the alerting state was enhanced for the upcoming stimuli only following the unpleasant odor stimulus. For the behavioral response results, classic ANT results associated with alerting cues emerged (Fan et al., 2002), that is, with higher RTs and error rates in no-cue conditions than in double-cue conditions (Williams et al., 2016; Yang and Xiang, 2019). These effects had greater advantages for the unpleasant odor condition than for the other two odor conditions. These findings are in line with previous ERP studies (Neuhaus et al., 2010; Galvao-Carmona et al., 2014; Williams et al., 2016; Yang and Xiang, 2019) and with behavioral studies (Bensafi et al., 2002b; Jacob and Wang, 2006; Boesveldt et al., 2010).

The alerting network in our study has been defined as phasic alertness by Posner (2008), as a momentary increment of alertness produced by warning signals. Fan et al. (2002) developed stimuli that could be preceded by a visual warning signal (a double asterisk cue) or by no signal. Our results are consistent with previous studies that found that the double cue was associated with a larger N1 amplitude than the no cue condition (Neuhaus et al., 2010; Williams et al., 2016; Yang and Xiang, 2019). Alerting is also considered an indicator of an individual’s sensitivity and readiness for new information (Petersen and Posner, 2012) and is a non-specific response outside of flexible control (Neuhaus et al., 2010). Our findings suggest that unpleasant odors may be a warning signal that enhances the innate alerting effect.

The results of some previous studies have suggested that pleasant odors, such as rosemary or peppermint, could lead to greater alertness as shown through behavioral and electrophysiological responses (Diego et al., 1998; Ilmberger et al., 2001; Moss et al., 2003). In our study, however, the unpleasant odor was associated with greater alerting-related behavioral and electrophysiological responses than the pleasant odor. These findings suggest that when an unpleasant odor is detected, more attention resources are mobilized and the alerting state is enhanced to face unknown dangers (Stevenson, 2010).

An enhanced alerting state supports the view of evolutionary psychology that the survival of organisms requires a faster response to unpleasant odors rather than to pleasant or neutral odors (Boesveldt et al., 2010). Because unpleasant odors often represent red flags, such as bad food or toxins (Dielenberg and McGregor, 2001; Boesveldt et al., 2010), the faster the response to a potential threat related to an unpleasant odor, the greater the possibility for survival and the ability to adjust to the next action (Mineka and Öhman, 2002). Mineka and Öhman (2002) argue that in human evolution, stimuli about threats and aversion are prioritized by our brains and require quick responses. The pleasantness of an odor significantly affects individual physiological arousal (Alaoui-Ismaïli et al., 1997; Bensafi et al., 2002c). The unknown threat factors that unpleasant odors may represent place the body at risk, and thus the body undergoes a stress response of enhanced mobilization of neural and hormonal networks to optimize cognitive, cardiovascular, immune, and metabolic functions to increase the chance of survival (Russell and Lightman, 2019). The stress response is partially reflected in reduced RTs and increased alertness.

As mentioned above, one of our main findings was that the presentation of an unpleasant odor caused a higher alerting state. In addition, however, pleasant odors also exhibited greater behavioral response effects than the odorless condition, suggesting that rosemary essential oil increased alerting (Diego et al., 1998; Moss et al., 2003). Although a pleasant odor enhanced alerting, an unpleasant odor had larger alerting effects on both behavioral and electrophysiological responses in our study.

### Orienting and Executive Control Networks and Odor Pleasantness

Our results assessing the orienting network indicated that behavioral and electrophysiological response differences were observed in only the cued condition, and that the odor condition was irrelevant to this. In previous studies using ANT, an orienting network effect under special task conditions has not been easy to obtain (Chang et al., 2015; Williams et al., 2016; Yang and Xiang, 2019). This may be due to methodological differences that restrain the overall results of an unpleasant odor effect on the orienting network, including the time between the odor stimulus presentation and performance of ANT, the time elapsed between the cue and target onsets (stimulus onset asynchrony), and the value of the spatial cue (Chang et al., 2015). Our data do not provide clear insights regarding this issue, and we encourage the exploration of this issue in future studies.

Our results also showed a larger conflict-associated N2 amplitude following the unpleasant odor presentation. However, this difference was not paralleled by a performance advantage, that is, an effect of the unpleasant odor on the behavioral response efficiency of the executive control network was insufficient (Chang et al., 2015). Hence, the results of the executive control network obtained in our study cannot be used as evidence of an improvement in effective performance associated with an unpleasant odor.

### Other Issues

Despite our behavioral and ERP results, in the topographic maps we present, we suggest that the frontal cortex also seems to play a role in the alerting network, while the occipital lobe also seems to be involved in the executive control network. Although this activation pattern has been shown in the topographic maps presented in previous studies (Neuhaus et al., 2010; Kratz et al., 2011; Chang et al., 2015; Donohue et al., 2016; Yang and Xiang, 2019), they have mainly focused on the activation of regions of interest (ROI) and ignored to explain the possible reasons of the activation of these non-ROIs. Hence, considering the inadequacy of previous studies on this issue, we argue that it is necessary to consider the particularity of ANT and the interactions among the three attention networks. For example, Chang et al. (2015) have suggested that ANT integrates measurements of the alerting network, orienting network, and executive control network and that studies focused on only one of the three attention networks are not fully comparable. Galvao-Carmona et al. (2014) also state that a careful assessment of the executive control network is required in ANT because ANT is a comprehensive measure for multiple attention networks, and there are interactions between them, which is consistent with the differences in the flanker congruency effects reported when assessing the alerting and orienting networks by Fan et al. (2009).

Our study did not detect differences in behavioral or electrophysiological responses associated with odor concentration, consistent with some previous studies (Bensafi et al., 2002a; Croy et al., 2013). However, other studies, for example, Jacob and Wang (2006), have reported that high-concentration odors are perceived faster than low-concentration odors, and other studies (Pause et al., 1997; Tateyama et al., 1998) have noted decreased latencies and increased amplitudes of early and late olfactory processing components with increased odor concentration. The effects of odor concentration continue to be debated. We suggest that one reason for the discrepant results may be the concentration values themselves, that is, whereas some studies assessed three concentration levels (i.e., high, medium, and low) (Covington et al., 1999; Croy et al., 2013), others assessed two levels (i.e., strong and weak) (Bensafi et al., 2002a). Thus, this dispute may be resolved in future studies by consideration of the concentration levels used, to obtain more persuasive evidence.

### Limitations and Future Directions

The present study had some limitations that should be considered when interpreting our results. First, the relatively low sample size has a partial negative effect on exploring the effect of odor concentration on the attention network. We used only two odors to explore the effect of odor pleasantness on attention networks, however, given the different effects of pleasant odors like lavender and rosemary oil on behavioral tests (Moss et al., 2003), is it possible that including more odors (either pleasant or unpleasant) will lead to different conclusions? Hence, further studies need to include more odors to explore this issue (Boesveldt et al., 2010; Croy et al., 2013; Deroy et al., 2013). In addition, the present study was conducted only in young men even though females have shown stronger odor identification, memory, and recognition than males (Brand and Millot, 2001; Oberg et al., 2002; Ferdenzi et al., 2013). Moreover, olfactory deterioration has been shown to occur in humans beginning in the fifth decade of life (Zhang and Wang, 2017), and older adults show reduced alerting compared with younger adults (Williams et al., 2016). Therefore, future studies should consider whether unpleasant odors show sex differences in ANT or whether the benefit of unpleasant odors may ameliorate attention alerting deterioration in older adults. Horii et al. (2013) reported the effect of exposure to a specific odor on the activity of the autonomic nervous system as well as reported that the efficiency of alerting is modulated by norepinephrine (Green et al., 2008). Thus, additional psychophysiology evidence is needed to explore the relationship between physiological responses or fluctuations and the effects on the attention network produced by odors. Finally, further spatial resolution information related to unpleasant odor and attention networks should be explored.

## Conclusion

The present study is the first, to our knowledge, to explore the effect of odor stimuli on multiple attention networks by assessing both odor pleasantness and odor concentration using an attention network task that assesses the three components of this network. Behavioral and electrophysiological response data analyzed jointly offer a greater wealth of information regarding the effects of odor stimuli on attention performance than either response explored alone. Thus, we found that not all factors related to odor stimulation, specifically here pleasantness and concentration, were associated with improved performance of the attention network. Rather we detected selective improvement by an unpleasant odor stimulation on the alerting state of the attention network. This finding supports ecological evidence for unpleasant odors acting as warning signals to help adult males alert to potential threats in the environment and provides cognitive performance evidence to support the relevant arguments of evolutionary psychology as well as situations in real-world daily life.

In summary, we provided behavioral and electrophysiological evidence that unpleasant odors significantly improve the alerting state in young adult males. Our results suggest that behavioral and electrophysiological indicators offer substantial contributions to understanding the effects of odor on complex attention networks.

## Data Availability Statement

The original contributions presented in the study are included in the article, further inquiries can be directed to the corresponding author/s.

## Ethics Statement

The studies involving human participants were reviewed and approved by the Ethics Review Committee of Shanghai University of Sport. The patients/participants provided their written informed consent to participate in this study.

## Author Contributions

MZ: substantial contribution to the conception, design and data analysis of the entire study, and drafting and revising the manuscript critically. XG: substantial contribution to the data acquisition and analysis, and documentation and format revision of the final manuscript version. JJ: substantial contribution to the data analysis and statistics analysis. XW: substantial contribution to the conception and design of the entire study, and revising the manuscript critically. All authors contributed to the article and approved the submitted version.

## Conflict of Interest

The authors declare that the research was conducted in the absence of any commercial or financial relationships that could be construed as a potential conflict of interest.

## Publisher’s Note

All claims expressed in this article are solely those of the authors and do not necessarily represent those of their affiliated organizations, or those of the publisher, the editors and the reviewers. Any product that may be evaluated in this article, or claim that may be made by its manufacturer, is not guaranteed or endorsed by the publisher.
